# Combining molecular evolution and environmental genomics to unravel adaptive processes of MHC class IIB diversity in European minnows (*Phoxinus phoxinus*)

**DOI:** 10.1002/ece3.650

**Published:** 2013-06-28

**Authors:** Helene Collin, Reto Burri, Fabien Comtesse, Luca Fumagalli

**Affiliations:** 1 Department of Ecology and EvolutionLaboratory for Conservation Biology, University of LausanneBiophore, 1015, Lausanne, Switzerland; 2 Institute of Integrative Biology, University of LiverpoolBiosciences Building, Crown St., Liverpool L69 7ZB, U.K; 3 Department of Evolutionary BiologyEvolutionary Biology Centre, Uppsala UniversityNorbyvägen 18D, 75236, Uppsala, Sweden

**Keywords:** 454 sequencing, AFLP, MHC class IIB, pathogens, *Phoxinus phoxinus*, selection

## Abstract

Host–pathogen interactions are a major evolutionary force promoting local adaptation. Genes of the major histocompatibility complex (MHC) represent unique candidates to investigate evolutionary processes driving local adaptation to parasite communities. The present study aimed at identifying the relative roles of neutral and adaptive processes driving the evolution of MHC class IIB (MHCIIB) genes in natural populations of European minnows (*Phoxinus phoxinus)*. To this end, we isolated and genotyped exon 2 of two MHCIIB gene duplicates (DAB1 and DAB3) and 1′665 amplified fragment length polymorphism (AFLP) markers in nine populations, and characterized local bacterial communities by 16S rDNA barcoding using 454 amplicon sequencing. Both MHCIIB loci exhibited signs of historical balancing selection. Whereas genetic differentiation exceeded that of neutral markers at both loci, the populations' genetic diversities were positively correlated with local pathogen diversities only at DAB3. Overall, our results suggest pathogen-mediated local adaptation in European minnows at both MHCIIB loci. While at DAB1 selection appears to favor different alleles among populations, this is only partially the case in DAB3, which appears to be locally adapted to pathogen communities in terms of genetic diversity. These results provide new insights into the importance of host–pathogen interactions in driving local adaptation in the European minnow, and highlight that the importance of adaptive processes driving MHCIIB gene evolution may differ among duplicates within species, presumably as a consequence of alternative selective regimes or different genomic context.

Using next-generation sequencing, the present manuscript identifies the relative roles of neutral and adaptive processes driving the evolution of MHC class IIB (MHCIIB) genes in natural populations of a cyprinid fish: the European minnow (*Phoxinus phoxinus*). We highlight that the relative importance of neutral versus adaptive processes in shaping immune competence may differ between duplicates as a consequence of alternative selective regimes or different genomic contexts.

## Introduction

In natural environments, heterogeneity in selection due to habitat fragmentation, climate fluctuations, and pathogen regime variation play a major role in shaping populations' genetic diversity, and eventually can lead to local adaptation (Kawecki and Ebert 43; Sommer 87; Barrett et al. 8; Gandon and Nuismer 33; Spurgin and Richardson 88). Pathogen communities can affect various fitness traits linked to immune resistance in hosts (Summers et al. 92; Simkova et al. 85; Evans and Neff 29). In return, hosts develop a wide range of immune responses to overcome pathogen infections (Acevedo-Whitehouse and Cunningham 1). Host–pathogen interactions are therefore ideal to study local adaptation, as both host and pathogen are subjected to varying selection in time and space, modifying their reciprocal fitness.

Genes of the major histocompatibility complex (MHC) are among the best candidates to study the genetics of the adaptive immune response (e.g., Klein 47; Kalz and Shykoff 42; Piertney and Oliver 72). MHC genes are the most polymorphic genes in vertebrates, and participate in the activation of the adaptive immune response (Klein 46). While MHC class I is involved in immune response against intracellular pathogens, class II triggers the immune response against extracellular pathogens. MHC polymorphism is believed to be mainly maintained by different mutually nonexclusive forms of pathogen-mediated balancing selection. First, heterozygote advantage confers a greater resistance to MHC-heterozygote individuals, as they are able to recognize a greater array of pathogen-derived antigens than homozygotes (Doherty and Zinkernagel 21; Hughes and Nei 39; Bernatchez and Landry 9; Garrigan and Hedrick 34). Second, under rare-allele advantage, rare alleles are selected for as they provide better resistance than common genotypes to which pathogens are adapted (Takahata and Nei 93; Bernatchez and Landry 9; Garrigan and Hedrick 34). Finally, pathogen communities may fluctuate over time and space, leading to temporally and spatially variable selection (Gandon and Nuismer 33; Spurgin and Richardson 88; Eizaguirre et al. 26). As a consequence, various subsets of alleles are selected across pathogen fluctuation cycles and thereby high MHC polymorphism is promoted. Also, phases of positive selection may alternate with phases where neutral processes are more important in shaping MHC polymorphism (Klein 47; Landry and Bernatchez 51). The null hypothesis that neutral processes (mutation, genetic drift, gene flow) shape MHC diversity can be tested by contrasting patterns of genetic differentiation between MHC genes and neutral markers. Under local adaptation to spatially heterogeneous pathogen pressures, populations are expected to be more genetically differentiated at MHC genes than at neutrally evolving genetic markers. Even though pathogen-mediated selection seem to be a major force driving genetic diversity at MHC genes, neutral processes can decrease genetic differentiation at putatively selected loci (Bernatchez and Landry 9; Van Oosterhout 97; Miller et al. 61). For instance, numerous cases reported bottleneck effects on MHC genes due to genetic drift in small and isolated populations (Miller et al. 61; Bollmer et al. 11; Spurgin et al. 89; Strand et al. 202). In these cases, genetic differentiation at MHC can be larger than at neutral loci as neutral genetic structure can be overestimated when population size is very low (Hedrick 37; Bernatchez and Landry 9; Van Oosterhout 97). Also, differentiation at MHC genes can be lower compared to neutral loci in the face of balancing selection (including cases of overdominance and heterozygote advantage) (Schierup et al. 78; Muirhead 64; Bernatchez and Landry 9; Blais et al. 10; André et al. 3; Bollmer et al. 11).

MHC class I and IIB genes have been well described in fish, especially in commercially important Salmonids like the Atlantic salmon (*Salmo salar*). This species frequently suffers from infectious diseases, such as the salmon anemia virus, or furunculosis caused by the bacteria *Aeromonas salmonicida*. The polymorphism observed at MHC class I and IIB is maintained through pathogen-mediated selection (Dionne et al. 19, 20) with particular alleles conferring resistance to furunculosis (Langefors et al. 52; Grimholt et al. 35). Other studies also reported a joint role of selection and neutral processes on the evolution of local adaptation at immune relevant genes in European trout (*Salmo trutta*) or Atlantic salmon (*S. salar*) (Landry and Bernatchez 51; Dionne et al. 19; Keller et al. 45). In comparison to *S. salar* and other Salmonids, cyprinid fish present a complex MHC architecture, with the presence of multiple duplicated MHC genes (Stet et al. 90; Shum et al. 84; Consuegra et al. 17; Seifertova and Simkova 80). More particularly, Simkova et al. (85) found a positive relationship between pathogen species richness and MHCIIB diversity in various other European cyprinids.

The European minnow (*Phoxinus phoxinus*) is a cyprinid occurring in most European freshwater systems. Local adaptation in growth and survival of *P. phoxinus* to a specific pathogen, the trematode *Diplostomum phoxini*, has been highlighted in the early 1990′s (Ballabeni and Ward 7; Ballabeni 6). Little is known about the influence of bacterial pathogens on this species' immunogenetics, especially on patterns of genetic structure and polymorphism at MHC loci. Recently, it has been shown that *P. phoxinus* displayed different body shape and adaptive genetic divergence to various abiotic factors such as lake-stream habitat, landscape topography, trophic dynamics, and geography, highlighting the capacity of this species to locally adapt to its environment (Collin and Fumagalli 16). The present study aims at identifying whether genes of the immune system (MHCIIB genes) contribute to local adaptation to pathogens in natural populations of European minnows. More specifically, we intended to identify local adaptation by (1) comparing patterns of genetic differentiation at MHCIIB exon 2 and neutral markers, and (2) identifying pathogen-mediated selection by testing the correlation of diversity of potentially pathogenic bacteria with MHCIIB polymorphism. To this end we characterized diversity of MHCIIB exon 2 in European minnows and bacterial 16S rDNA in water samples using 454 amplicon sequencing. We identified two MHCIIB gene duplicates (DAB1 and DAB3) both presenting signs of historical balancing selection, but displaying contrasted patterns of adaptive evolution. Indeed, DAB3 duplicate exhibited evidence for pathogen-mediated selection contrarily to DAB1 duplicate, demonstrating that neutral and adaptive processes' roles differ among MHCIIB duplicates in European minnows.

## Material and Methods

### Sampling and DNA extraction

**Figure 1:**
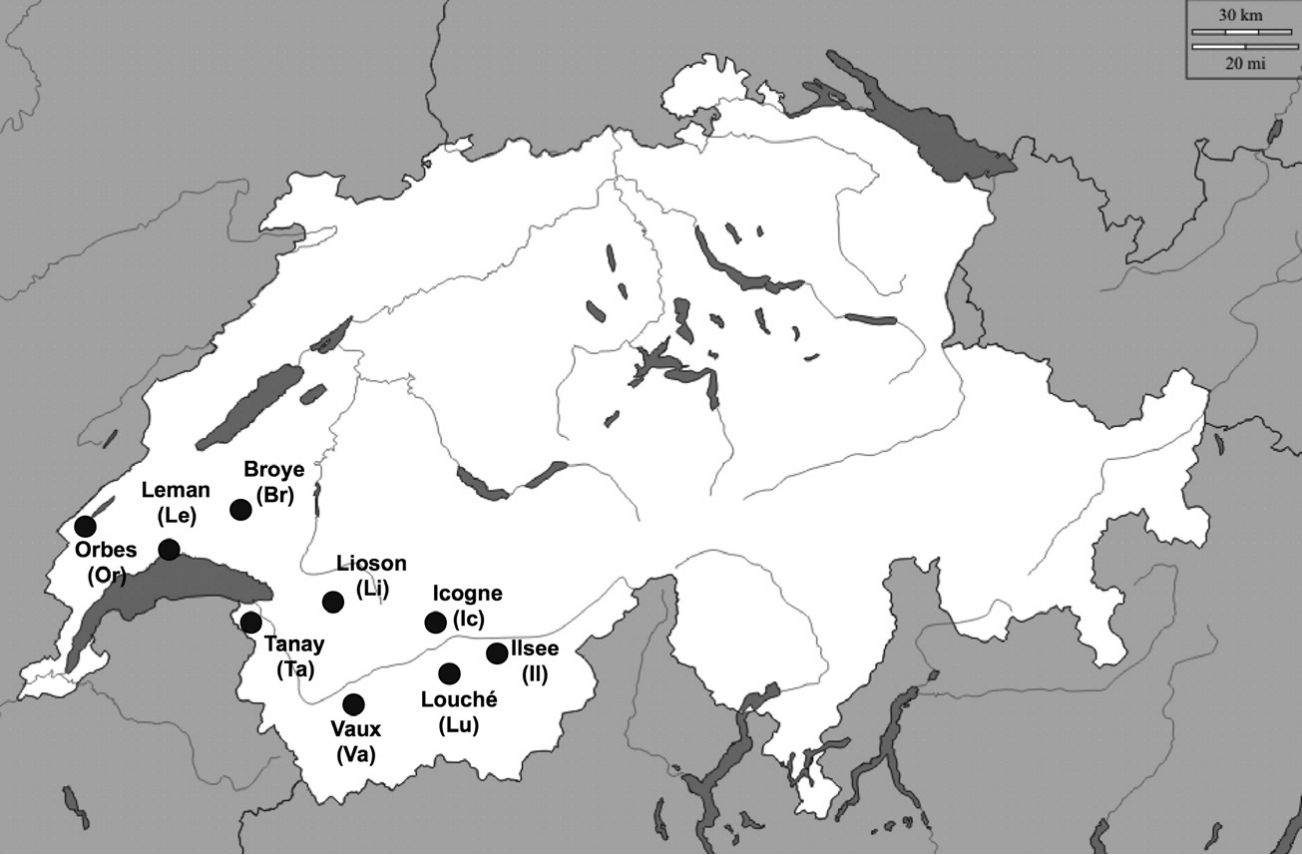
Map showing the locations of the nine populations sampled in the South Western Swiss Alps. Population abbreviations are indicated in brackets.

One hundred and seventy-six European minnows were collected in nine populations from the Western Swiss Alps (Fig. 1) during summer 2008 and 2009, when bacteria communities are the most diverse. Adult fish were killed using AQUI-S® (AQUI-S New Zealand Ltd, Lower Hutt, NZ) and immediately stored in absolute ethanol for further genetic analyses. To assess bacterial diversity for each population, water samples were collected in 1 L sterile bottles and filtered using autoclaved membrane filters (0.2 μm). Filters were stored in 3 mL of lysis buffer (0.75 mol/L Sucrose, 400 mmol/L NaCl, 20 mmol/L EDTA (pH 8.0), 50 mmol/L Tris-HCl (pH 9.0) at −80°C. Filters in lysis buffer containing bacterial DNA were digested overnight at 37°C with sodium dodecyl sulfate (1%), proteinase K (60 μg mL), and CaCl_2_ (90 mmol/L). Bacterial DNA was extracted from 500 μL of digested lysate using phenol chloroform (pH 7.5), sodium acetate (15 μmol/L), isopropanol, and ethanol precipitation procedure. DNA was resuspended in sterile water and concentration was adjusted to 10 ng μL^−1^. DNA from fish was extracted using the Gentra Puregene Qiagen kit according to the manufacturer instructions. DNA concentration was adjusted to 50 ng μL^−1^.

### MHCIIB isolation and characterization

In order to isolate and characterize MHCIIB exon 2 of *P. phoxinus,* a subset of four individuals from six populations (Ta, Ic, Li, Il, Va, Lu) were used. We first amplified the 3′-end of exon 2, intron 2 and the 5′-end of exon 3 using the primers FishC12S and Fish12R from Ottova et al. (69) (for primer description and sequences, see Fig. S1 and Table S1). Additionally, intron 1 and the 5′-end of exon 2 were amplified using the reverse complement of FishC12S (FishC12S-R), and a newly designed primer in exon 1 (FishEx12F-bis). The latter primer was designed in conserved regions of exon 1 from *Cyprinus carpio*,* Barbus intermedius,* and *Danio rerio* sequences (Van Erp et al. 96; Kuroda et al. 50; Kruiswijk et al. 49). We then designed species-specific primers amplifying the entire exon 2, which was included in *P. phoxinus* the two duplicates DAB1 and DAB3 (inferred from phylogenetic analyses with other cyprinid MHCIIB exon 2 sequences, see below). PCR for the two duplicates of MHCIIB exon 2 characterization were performed in a 25 μL reaction volume containing 1.5 mmol/L MgCl_2_, 1× PCR buffer (Qiagen), 0.4 μ mol/L of each primer, 100 μ mol/L of each dNTP, 1 unit of Taq Polymerase (Qiagen), and 50 ng of genomic DNA. Thermal cycling was carried out on a Biometra T Professional Standard Thermocycler (Biometra, Goettingen, Germany), with an initial denaturating step of 95°C for 3 min, followed by 39 cycles of 30 sec of denaturing at 95°C, 1 min annealing at 52°C and 1 min 30 sec of extension at 72°C, and a final extension of 5 min at 72°C. PCR products were cloned into the pGEM T-easy vector (Promega, Madison, WI). Between four to eight positive clones for each duplicate of MHCIIB exon 2 were purified with the Wizard SV PCR clean-up system (Promega) and sequenced using the Big Dye 3.1 Terminator cycle sequencing kit (Applied Biosystems, Carlsbad, CA) on a ABI 3100 PRISM genetic analyzer (Applied Biosystems) using SP6 and T7 standard primers and universal procedures. Sequences from clones were read using BioEdit 7.09 (Hall 36). In order to ensure that the two duplicates corresponded to functional genes and not pseudogenes, we also amplified *P. phoxinus* cDNA following the protocol mentioned above (Text S1). Start and end of each exon 2 duplicates were deduced by comparison with known cDNA and gDNA sequences of *D. rerio* (Kuroda et al. 50).

### 454 sequencing of MHCIIB exon 2 and 16S bacterial rDNA

In order to amplify the two duplicates of MHCIIB exon 2 and bacterial 16S rDNA in a partial 454 GS FLX Titanium run (Roche, Branford, CT), we pooled 18–20 individuals per population and randomly assigned each population five base pair tagged PCR primers (Table S1, Fig. S1). These primers were then used in locus-specific PCRs to amplify DAB1 and DAB3. The same tagging strategy was used to amplify bacterial 16S rDNA from the nine populations. PCRs for both MHCIIB exon 2 and 16S rDNA were conducted on Biometra T Professional Thermocyclers (Biometra) in a final volume of 25 μL, using 1 × Q-solution (Qiagen), 1× buffer (Qiagen), 1.5 mnol/L MgCl2 (Qiagen), 0.2 mmol/L dNTPs (Peqlab, Erlanger, Germany), 0.2 μmol/L of each primer, 2 units of Taq (Qiagen), and 20 ng of genomic DNA. Cycling conditions included an initial denaturation step at 95°C for 3 min followed by 35 cycles at 95°C for 30 sec, annealing at 52°C (DAB1 and DAB3 loci) or 58°C (bacterial 16S rDNA) for 1 min, 72°C for 1.5 min, and a final extension step at 72°C for 5 min. PCR product concentrations were estimated simultaneously on agarose gel and with a Nanodrop spectrometer (Nanodrop Technologies, Wilmington, DE). PCR products with similar concentrations were pooled and purified using the MinElute PCR Purification kit (Qiagen). Purified PCR products' concentrations were estimated a second time in order to produce a final pool with equimolar quantities of PCR products (Babik et al. 5; Kloch et al. 48). Final pool concentration was adjusted to 20 ng μL^−1^ in a final volume of 30 μL and sequenced on a partial 454 GS FLX Titanium run (Microsynth AG, Switzerland).

### Sequence retrieval and filtering

#### Bacterial 16S rDNA data

The 16S rDNA multifasta file resulting from the 454 run was sorted according to tag and primer sequences. In order to attribute each bacterial 16S rDNA sequence to a taxonomic unit (genus or species in our case), 16S rDNA sequences were submitted to the Bayesian Ribosomal Database Project (RDP) Classifier (http://rdp.cme.msu.edu/classifier/classifier.jsp; Wang et al. 100; Cole et al. 15). Sequences shorter than the expected amplicon size of 250 bp and with a bootstrap support inferior to 50% for taxon assignment were discarded to guarantee sufficient sequence length to reach a high taxonomic resolution and accuracy, respectively. A community data matrix containing the presence–absence of each species per population was built. We identified bacteria potentially pathogenic in fish using the classification established by Austin and Austin (4).

#### MHCIIB data

The primer used to sequence DAB1 and DAB3 using 454 technology amplify 276 bp approximately (Fig. S1). Altogether with primers and tag, read length exceeded the length that 454 run cold sequences, resulting in reads whose length was shorter than expected. All sequences were thus truncated to 220 bp in order to maximize the number of sequences for further analyses (Spurgin et al. 89). Sequences from the multifasta DAB1 and DAB3 files containing a complete tag and a match to the forward or reverse primer were retained for further analysis. From these files we identified authentic MHCIIB variants (true variants or TV) according to several criteria, partially following Galan et al. (32). First, we counted for each population the number of unique variants (all sequences differing in at least one base pair or one indel). We then removed variants appearing only once, or in less than two populations in order to eliminate artifactual variants (AV) resulting from polymerase errors and sequencing errors. This step might remove some rare alleles, but avoids including AV in further analyses. As in the four to eight cloned sequences from four test populations we never observed frameshift mutations and because here we are exclusively interested in functional MHCIIB variation, the remaining sequences were aligned using ClustalW (Thompson et al. 95) in Mega 5.0 (Tamura et al. 94) and sequences with indels whose lengths differ from a multiple of three were excluded. Even though PCR products were included in equimolar quantities for sequencing, the number of variants represented only semiquantitative data, and the number of reads per TV might not be representative of the actual TV number in the data set. For further analyses, we thus decided to use a presence–absence matrix of TV instead of allele frequencies.

### AFLP data

In order to compare patterns of genetic differentiation at neutral markers with those observed at MHCIIB, we generated amplified fragment length polymorphism (AFLP) markers using the same protocol as described in Collin and Fumagalli (16). AFLP were automatically genotyped using Genemapper version 4.0 software (Applied Biosystems) and genotypes were manually cross-validated twice. To avoid genotyping errors and a confounding factor from genotyping plates, 38 individuals from different populations were replicated independently thrice, and all individuals within and between populations were randomized across plates from the extraction step on. All loci that were not 100% reproducible across the 38 individuals were removed from the analysis. For data analysis, populations were assumed to be under Hardy–Weinberg equilibrium and nonpolymorphic (noninformative) loci were removed, resulting in 1′930 polymorphic loci. As we were interested in comparing neutral genetic divergence with MHCIIB divergence between populations, we excluded loci potentially under selection. The latter were inferred using BAYESCAN 1.0 (Foll and Gagiotti 31) following a method previously developed specifically for European minnows (Collin and Fumagalli 16). This software implements two models (including and not including selection, respectively) using reversible jump Markov Chain Monte Carlo sampling. It then estimates the posterior probability for a locus to be under selection, depending on a detection level that we set to “substantial” corresponding to a posterior probability superior to 0.76. The choice of this specific detection level is arbitrary, but has been previously demonstrated to not affect conclusions (Collin and Fumagalli 16). Also, choosing a “substantial” detection level allows the detection of loci under very weak selection to generate a fully “neutral” data set with presence–absence of 1′665 neutral AFLP loci per population.

### Statistical analyses

#### Bacteria diversity

In order to explore nonpathogenic and pathogenic bacterial composition between sampling locations, we conducted a permutational multivariate analysis of variance based on dissimilarities, that is, beta nonpathogenic and pathogenic bacteria diversity (ADONIS), using populations as a factor. Betadiversity was calculated using the nonpathogenic bacteria and pathogens' abundance matrices (excluding two populations, Br and Ta, as no pathogenic bacteria were recorded in these two locations). We finally conducted a canonical correspondence analysis (CCA) in order to analyze the correlation between pathogen composition and various environmental factors characterizing each sampling location and which have been shown to influence bacteria distribution (Yannarell et al. 106; Lindström et al. 55; Yannarell and Triplett 105; Newton et al. 201; Shade et al. 83; Lindström and Langenheder 54) (dissolved oxygen, altitude, landscape slope, soil pH, mean number of annual frost days, primary productivity; see Collin and Fumagalli 16 for a full description of environmental variables). All analyses were conducted in R using the “vegan” package (Oksanen et al. 68).

#### MHCIIB diversity

We were able to amplify 55 codons (165 bp) for DAB1 and 58 codons (174 bp) for DAB3, in which peptide-binding region (PBR) represented the most polymorphic codons as they bind pathogenic antigens (Hughes and Yeager 40). We inferred the locations of PBR and non-PBR regions from human MHCIIB molecular structure (Brown et al. 12). Two types of indices were estimated for MHCIIB loci. First, amino acid diversity at every codon position was estimated separately for DAB1 and DAB3 using DIVAA (Rodi et al. 76). Then DAB1 and DAB3 amino acid polymorphism was measured as the percentage of polymorphic codons for PBR, non-PBR and all codons, respectively. We tested for differences between DAB1 and DAB3 amino acid polymorphism at PBR, non-PBR, and all codons using a t-test. The relative rate of synonymous (*d*_S_) and nonsynonymous (*d*_N_) substitutions was calculated according to Nei and Gojobori (66) using MEGA 5.0 (Tamura et al. 94). MEGA 5.0 was also used to perform a *Z*-test of selection at all amino acid positions, PBR and non-PBR sites, under the null hypothesis that *d*_N_ = *d*_S_. Additionally, we tested for positive selection using a maximum likelihood method implemented in CODEML (PAML v4.3 package; Yang 102). Phylogenetic tree topologies were reconstructed using MrBayes 3.1 (Ronquist and Huelsenbeck 77) for DAB1 and DAB3 duplicates separately using *Squalius cephalus* as outgroup (Genbank accession number HQ595117 for DAB1 and HQ595146 for DAB3). We then used CODEML to implement four site models: two neutral models (M1a and M7) and two models allowing for positive selection (M2a and M8) (Yang et al. 103). The models were compared using likelihood ratio tests statistics (LRT statistics) calculated as two times the difference between the Log-likelihoods of the models allowing for selection and the neutral models. LRT statistics were compared to a chi-square distribution with 2 degrees of freedom. Positively selected codons were identified using the Bayes empirical Bayes (BEB) approach described in Yang et al. (104).

#### MHCIIB and bacteria diversity

In order to provide evidence for pathogen-mediated selection acting on particular MHC sites, we tested the null hypothesis of no difference in the correlation between MHCIIB polymorphism and bacterial pathogen diversity (Dionne et al. 19). We calculated species richnesses of pathogenic bacteria, nonpathogenic bacteria, and overall bacterial species richness per population using the R library “vegan” (Oksanen et al. 68). We then analyzed the relationship between the percentage of amino acid polymorphism at PBR, non-PBR codons, and bacterial species richness (pathogenic, nonpathogenic, overall) using linear models. Significance was tested using analysis of variance (ANOVA).

#### Genetic differentiation at MHCIIB and neutral AFLPs

As our study includes population-based and not individual-based data, we used dissimilarity indices, that is, Euclidean distances between pairs of populations as an estimate for population differentiation. First, Euclidean distance matrices were estimated based on (1) TV presence–absence in each population for DAB1 and DAB3 and (2) AFLP band presence–absence in each population. DAB1, DAB3, and AFLP distance matrices were then standardized to a 0/1 scale using the following formula: (*d*_x_−*d*_min_)/(*d*_max_−*d*_min_), where d_x_ represents the pairwise Euclidean distance between two populations, *d*_max_ the maximum Euclidean distance between two populations, and *d*_min_ the minimum Euclidean distance between two populations identified in the matrices. We first tested for isolation by geographical distance (IBD) with Mantel tests both for neutral AFLP and MHCIIB (separately for DAB1 and DAB3). In order to estimate if MHCIIB differentiation exceeds neutral differentiation along geographic clines, we tested whether isolation by distance (IBD) patterns in MHCIIB persisted when accounting for neutral population differentiation using a partial Mantel test. We tested for correlation between differentiation at AFLP and at MHCIIB loci (DAB1 and DAB3 separately) using a Mantel test in order to highlight a potential effect of divergent selection which would increase levels of genetic differentiation at immune loci compared to neutral markers. We also aimed at obtaining the full neutral distribution of IBD slopes, in order to directly compare this distribution to IBD slope values observed at the MHC, hence providing a cross-test of IBD not only using neutral population-based data but individual neutral data. We estimated individual-based AFLP Fst matrices between pairs of populations using a subset of randomly chosen AFLP markers among the 1665 available. The regression slopes between *F*_st_ matrices and geographical distance matrices were obtained from Mantel tests. We then compared the distribution of neutral regression slopes (minimum and maximum values of *R*^2^ obtained with AFLP markers) with values obtained from MHC loci. All Mantel and partial Mantel tests were implemented with 1000 permutations in each case using the “vegan” library in R (Oksanen et al. 68).

## Results

### MHCIIB characterization and diversity

All MHCIIB sequences obtained by cloning four individuals of six *P. phoxinus* populations blasted against previously published MHCIIB sequences of other cyprinids (including *Abramis brama, Barbus bocagei, B. intermedius, Brachydanio rerio, C. carpio, Ladigesocypris ghigii, Rhodeus ocellatus,* and *S. cephalus*) and salmonids (*S. salar*), with sequence similarities ranging from 79% to 92%. As reported also from other species (Ottova et al. 69; Seifertova and Simkova 80), we identified two functional duplicates, corresponding to DAB1 and DAB3. Functionality of the genes was supported by successful cDNA amplification (Text S1), and orthology with DAB1 and DAB3 of other cyprinids was confirmed by two well-separated paralog sequence clusters in a phylogenetic analysis (Appendix 1), with alleles from different species found in each duplicate cluster. We thus further studied two MHCIIB paralogs, with large differences in intron 1 and 2 lengths between the two groups of sequences strengthening the evidence for at least two gene copies (data not shown). A number of clues such as (1) successful amplification of cDNA (2) the absence of stop codon or frameshift mutations (3) the presence of many features characteristic of functional MHCIIB genes (N-linked glycosylation sites and the two conserved cysteine residues) are consistent with a functional antigen binding protein.

454 sequencing produced 7′591 DAB1 and 7′877 DAB3 reads (Text S2 and S3). Average, minimum and maximum read lengths and quality scores frequencies are reported in Appendix 2 and Fig. S2. The first step consisting in the identification of unique TV and AV resulted in 3′456 retained sequences for DAB1 and 3′811 for DAB3. After removing sequences appearing only once or less in the two populations, 162 variants for DAB1 and 177 for DAB3 were left. Sequences were aligned and partial intronic sequences were removed. After alignment and removal of sequences with frameshift indels, 19 TV were found for DAB1 (956 sequences in total, 13% of the initial reads number) and 30 TV for DAB3 (1′046 sequences in total, 13% of the initial reads number).

For both DAB1 and DAB3, amino acid diversity was highest at the 10 (DAB1) and 11 (DAB3) codons matching human PBR codons (Appendix 3). Mean DAB1 and DAB3 amino acid diversity at PBR codons were 0.117 and 0.139, respectively, and were significantly lower at non-PBR sites (0.009 and 0.059 for DAB1 and DAB3, respective *t*-tests: df = 8.832, *t* = −3.685, *P = *0.005; df = 10.438, *t* = −4.219, *P = *0.002). In concordance with these results, the *Z*-test of selection showed evidence for diversifying selection on PBR codons as the rate of nonsynonymous substitution (*d*_S_) over synonymous substitution (*d*_N_) was significantly higher than expected under neutral expectations (Table 1). We tested for differences in amino acid polymorphism between DAB1 and DAB3 duplicates. The t-tests revealed that DAB3 presented greater amino acid polymorphism only at PBR codons (*t* = 2.402, df = 15.887, *P = *0.014). The maximum likelihood method implemented in CODEML revealed that models allowing for positive selection (M2a and M8) better explained DAB1 and DAB3 sequences evolution than neutral models (M1a and M8) (Table 2). Models M2a and M8 identified 13 and 14 codons, respectively, as having evolved under positive selection in DAB1, with, respectively, seven and eight of these corresponding to human PBR sites. For DAB3, five codons were inferred having evolved under positive selection, all of them corresponding to PBR sites.

**Table 1:** Ratio of synonymous (*d*_S_) to nonsynonymous (*d*_N_) substitutions for the two duplicates of MHC class IIB (MHCIIB) exon 2 (DAB1 and DAB3)

	DAB1				DAB3						
Region	Codon	*d*_N_/*d*_S_	*P*	Codon	*d*_*N*_ */d*_*S*_	*P*
Non-PBR	45	0.664	0.508	47	0.548	0.585
PBR	**10**	**4.320**	**<0.001**	**11**	**7.038**	**<0.001**
Total	**55**	**2.131**	**0.035**	58	1.240	0.217

Bold font indicates significant departure from neutrality test at *P* < 0.05.

**Table 2:** Evidence of positive selection on MHC class IIB (MHCIIB) exon 2 duplicates

Duplicate	Model	Ln *L*	Parameter estimates	Positively selected sites	LRT statistic
DAB1	M1a	−807.21	*p* = 0.548 (p_1_ = 0.452), ω = 0, ω_1_ = 1	Not allowed	
	M2a	−775.92	p_0_ = 0.501, p_1_ = 0.144 (p_2_ = 0.355), ω = 0, ω_1_ = 1, ω_2_ = 9.121	6, 8, 10, 14, 22, 24, 25, 35, 36, 44, 45, 51, 54	62.584 (*P *<* *0.001)
	M7	−807.43	*p* = 0.005, q = 0.005	Not allowed	
	M8	−775.93	p_0_ = 0.643 (p_1_ =0.357) *p* = 0.005, q = 0.020, ω = 8.965	6, 8, 10, 14, 22, 24, 25, 28, 35, 36, 44, 45, 50, 51, 54	62.991 (*P *<* *0.001)
DAB3	M1a	−1015.73	*p* = 0.663 (p_1_ = 0.337), ω = 0.081, ω_1_ = 1	Not allowed	
	M2a	−971.28	p_0_ = 0.454, p_1_ = 0.457 (p_2_ = 0.089), ω = 0.065, ω_1_ = 1, ω_2_ = 9.402	8, 10, 24, 26, 28	88.906 (*P *<* *0.001)
	M7	−1019.09	*p* = 0.159, q = 0.221	Not allowed	
	M8	−971.60	p_0_ = 0.911 (p_1_ = 0.089) *p* = 0.023, q = 0.020, ω = 9.535	8, 10, 24, 26, 28	94.963 (*P *<* *0.001)

Sites inferred as being under positive selection with a posterior probability >0.99 are also reported as numbers corresponding to their position in Fig. S4. LRT statistics were used to compare (1) M1a and M2a models and (2) M7 and M8 models to a chi-square distribution (df = 2). Log-likelihood and parameter estimates calculated by CODEML are presented.

### Genetic differentiation and diversity at MHCIIB and neutral AFLPs

#### Isolation by distance

**Figure 2:**
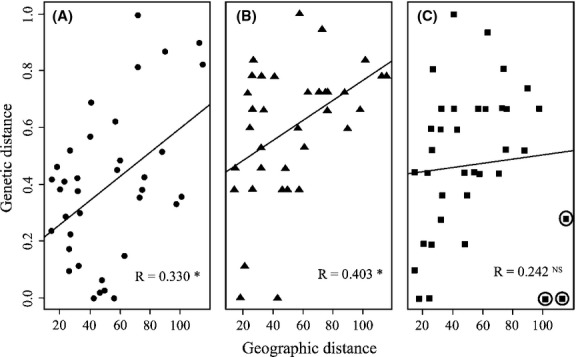
Isolation by distance patterns for genetic differentiation between populations at neutral AFLP (A), DAB1 (B), DAB3 (C). Lines represent the least square regression between genetic distance and geographical distance. R statistics from Mantel tests and their significance (* significant at 95% level, ^NS^ non-significant at the 95% level) are indicated. The three outlier pairs of populations breaking down the isolation by distance (IBD) pattern at MHC class IIB (MHCIIB) DAB3 are circled in black (C).

Isolation by distance was observed at neutral loci and DAB1 (Fig. 2; Mantel test respectively, *R* = 0.330, *P = *0.037 and *R* = 0.403, *P = *0.025). DAB3 showed a tendency of IBD (Mantel test, *R* = 0.242, *P = *0.086). The nonsignificance of this correlation was driven by three outlier population pairs (Ic-Or, Il-Or, Lu-Or) for which low levels of genetic differentiation was not explained by the geographic distance separating them. The three outlier population pairs are highlighted by black circles in Fig. 2C. Removing them resulted in significant IBD (Mantel test, *R* = 0.515, *P = *0.004). The pattern of IBD at DAB1 persisted when controlling for neutral genetic differentiation (*R* = 0.297, *P = *0.042), and stayed nonsignificant for DAB3 (*R* = 0.188, *P = *0.140). Again, when removing the three outlier pairs of populations and controlling for neutral differentiation, significant IBD was found also at DAB3 (*R* = 0.459, *P = *0.014).

#### Patterns of genetic differentiation at MHCIIB and neutral AFLP

**Figure 3:**
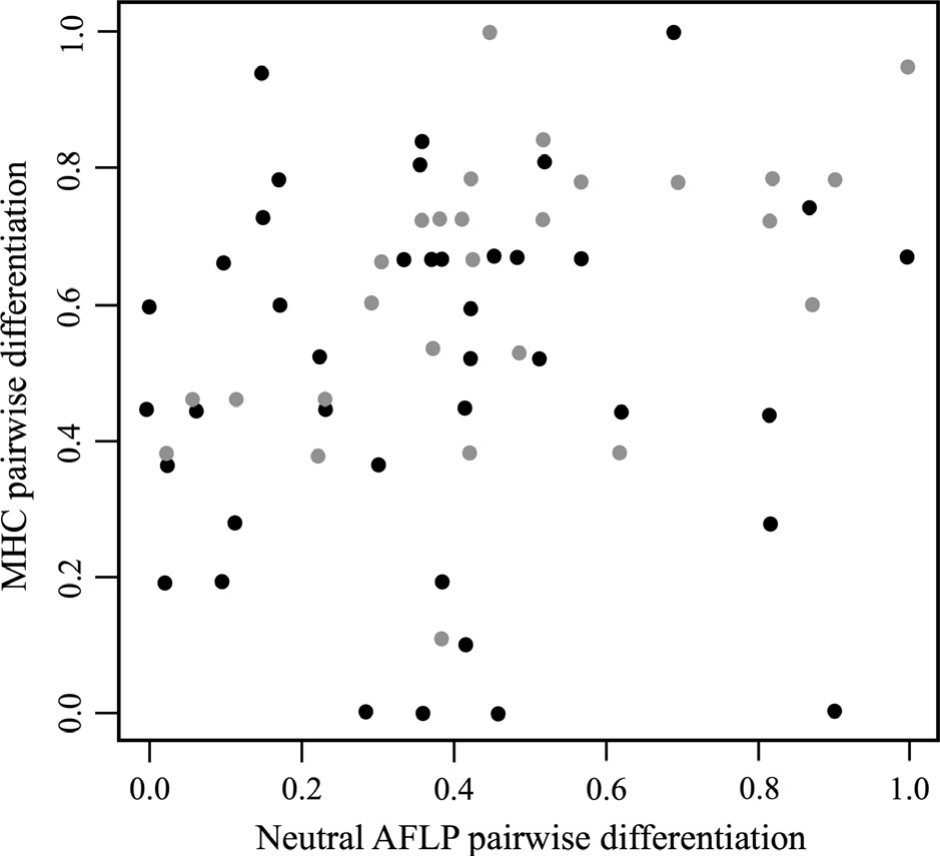
Correlation between the extent of genetic differentiation at neutral AFLP loci and MHC class IIB (MHCIIB) loci (DAB1: gray closed dots, Mantel test: *R* = 0.469, *P = *0.006; DAB3: black closed dots Mantel test: *R* = 0.207, *P = *0.137).

Patterns of differentiation at neutral and MHCIIB duplicates were significantly correlated for DAB1, but not for DAB3 (Fig. 3; Mantel test; DAB1, *R* = 0.469, *P = *0.006 and DAB3, *R* = 0.207, *P = *0.137). Again, removing the three outlier pairs of populations resulted in significantly correlated genetic differentiation at AFLP markers and DAB3 (Mantel test; *R* = 0.313, *P = *0.062). The neutral distribution of regression slopes ranged from −0.263 to 0.839, which includes values observed at MHC loci (0.445 and 0.242 for DAB1 and DAB3, respectively).

### Bacterial and MHCIIB diversity

**Figure 4:**
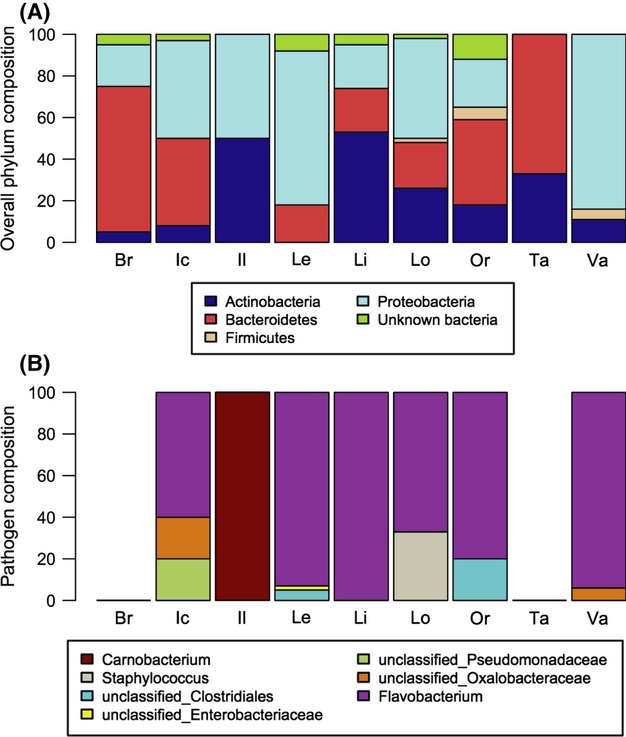
Barplot of 16S rDNA sequences relative abundance of phyla (A) and pathogen species (B) for sampling location (full population names and abbreviation are detailed in Figure 1).

**Figure 5:**
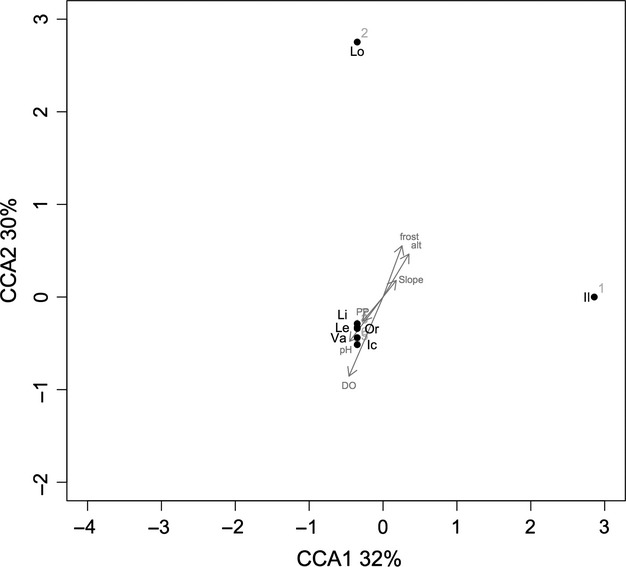
Canonical correspondence analysis correlation biplot showing the relationship between pathogen abundance and composition per sampling location (for full names, see Fig. 1) and environmental variables (DO, dissolved oxygen; alt, altitude; Slope, landscape slope; pH, soil pH; frost, mean number of annual days of frost; PP, primary productivity). Explanatory environmental variables are represented by gray arrows whose length indicated the strength of correlation between variables and pathogen's ordination score. Angles between variables reflect their degree of correlation. Gray numbers represent each pathogen species (1, *Carnobacterium*; 2, *Staphylococcus;* 3, *unclassified Clostridiales;* 4, *unclassified Enterobacteriaceae;* 5, *unclassified Pseudomonaceae;* 6, *unclassified Oxalabacteraceae;* 7, *Flavobacterium*).

To investigate how pathogenic bacteria affect *P. phoxinus* populations' MHCIIB diversity at a local scale during summer, we first characterized pathogenic and nonpathogenic bacterial communities for each population using 454 amplicon sequencing of 16S rDNA. Classification in the Ribosomal Database Project retained sequences with a length >200 bp resulting in a total of 374 sequences of 16S bacterial rDNA out of 548 reads (see Appendix 2 for read details). Based on Bergey's nomenclature used by the classifier taxonomic assignment tool implemented in RDP, 41 different species were identified in the nine sampling sites analyzed, and 40 other species were not determined and thus treated as being different species in the data set. Seven potentially pathogenic species were identified following Austin and Austin (4): *Lactobacillus sp*. (Carnobacterium), *Staphylococcus sp*., *Flavobacterium sp*., unclassified *Clostridiales*, unclassified *Enterobacteriaceae*, unclassified *Pseudomonadaceae,* and unclassified *Oxalobacteraceae*. We therefore partitioned the bacteria into three different data sets: “pathogenic bacteria,” “nonpathogenic bacteria”, and “all bacteria.” The species richness index calculated for these three data sets ranged from zero to three for pathogens, from two to 36 for nonpathogens, and from three to 39 for all bacteria. We examined the overall bacteria phylum composition for each sampling location (Fig. 4A). We found four main phyla across the nine sampling locations screened: *Actinobacteria*,* Bacteroidetes*,* Firmicutes,* and *Proteobacteria*. A small proportion of unknown bacteria were recorded. Il and Va locations were characterized by the absence of the *Bacteroidetes* phylum and Ta by the absence of *Proteobacteria*. *Firmicutes* was the less abundant phylum in all sampling locations. No pathogenic bacteria were recorded at Br and Ta locations (Fig. 4B). Il location was characterized by *Carnobacterium* (*Firmicute*), while in other locations *Flavobacterium* pathogens (*Bacteroidetes*) predominated (Fig. 4B). ADONIS analysis revealed no significant difference in pathogenic and nonpathogenic bacteria composition between locations. CCA highlighted that 62% of the variance observed in the multivariate data set (pathogen diversity and environmental variables for each location) were explained by CCA1 and CCA2 axes (Fig. 5). As mentioned previously, Il and Lo locations were differentiated from the others as their pathogen distribution (1) was rather different, dominated by *Carnobacterium* and *Staphylococcus*, respectively, and (2) was positively associated to higher altitudinal sites and topography. Pathogen compositions at other sampling locations were characteristic of highly productive (eutrophic) sites at lower elevation.

We used linear models and ANOVAs to explore the relationship between amino acid polymorphism at MHCIIB and species richness of pathogenic bacteria, nonpathogenic bacteria, and all bacteria. The percentage of polymorphic amino acid sites for the two MHCIIB duplicates ranged from 80% to 100% for DAB1, 82% to 100% for DAB3 at PBR codons, and at non-PBR codons from 20% to 27% for DAB1 and 19% to 36% for DAB3. Only the percentage of polymorphic amino acid sites within PBR codons at DAB3 showed significant positive association with pathogen species richness (ANOVA: df = 7, *F* = 12.015, *P = *0.010). ANOVA results of linear regression models are summarized in Table 3.

**Table 3:** General linear model results regarding the effects of bacterial richness (nonpathogen and pathogen) on the percentage of polymorphic amino acid sites for the two MHCIIB duplicates (DAB1 and DAB3)

	PBR	Non-PBR	PBR	Non-PBR
All bacteria (df = 7)	*F* = 0.536 *P = *0.488	*F* = 0.355 *P = *0.570	*F* = 0.440 *P = *0.528	*F* = 3.488 *P = *0.104
Nonpathogen (df = 7)	*F* = 0.740 *P = *0.418	*F* = 0.460 *P = *0.521	*F* = 0.244 *P = *0.637	*F* = 4.142 *P = *0.081
Pathogen (df = 7)	*F* = 0.532 *P = *0.489	*F* = 0.148 *P *= 0.712	***F***** = 12.015** ***P***** = 0.010**	*F* = 0.015 *P = *0.905

Bold font indicates a significant effect at *P* < 0.05.

## Discussion

The present study aimed at examining whether the cyprinid fish *P. phoxinus* exhibited MHCIIB-related local adaptation to potentially pathogenic bacterial communities to which fish populations are exposed to. Our results suggest pathogen-mediated local adaptation in European minnows at two MHCIIB loci (DAB1 and DAB3), though they appeared to be driven by different aspects of pathogen-mediated selection. Patterns of genetic differentiation at DAB1 suggest that selection favored different alleles in each population, resulting presumably in better immune defense against local pathogens. This pattern was also found for DAB3, but only partially when removing the three pairwise comparisons with largest geographic distance. Contrary to its paralog, genetic diversity at DAB3 was correlated with local pathogen diversities. This suggests that although locally adapted alleles were favored by selection also at DAB3, selection for locally adapted genetic diversity overpowered the selection for locally adapted alleles. The interplay between these different selection processes may explain the breakdown of the overall pattern of IBD at DAB3.

### Bacterial community composition

In the present study, pyrosequencing provided a fast way to characterize bacterial communities with a greater sequencing depth than any other method (Sogin et al. 86). We found common phyla of freshwater lakes, such as *Bacteroidetes*,* Actinobacteria,* and *Proteobacteria* (Newton et al. 67). We found seven potentially pathogenic bacteria species that were inferred from multiple previous studies as driving factors of fish diseases (Austin and Austin 4; Isik et al. 41; Clements et al. 13, 14; Moyer and Hunnicutt 63; Embar-Gopinath et al. 28; Loch et al. 56; Sekar et al. 81; Von Siebenthal et al. 99; Prasad and Kumar 73; Newton et al. 67; Mouchet et al. 62). Their composition didn't significantly differ between locations, but was influenced by various environmental factors such as altitude, landscape topography, and productivity. These factors have been previously reported to play a role in bacteria species distribution and abundance at local and regional scales (Yannarell et al. 106; Lindström et al. 55; Yannarell and Triplett 105; Newton et al., 201; Shade et al. 83; Lindström and Langenheder 54). No pathogenic bacteria were recorded at two locations (Br and Ta), suggesting that other types of pathogens may be present and play a role in shaping MHC genetic diversity in these populations. Even though ADONIS analysis showed little differences in pathogen composition, the CCA analysis revealed a high heterogeneity in pathogen distribution between sites, especially for Il and Lo. Il was dominated by *Carnobacterium*, which was reported as a major fish pathogen associated with warm water streptococcosis (Michel et al. 60; Mata et al. 59; Leisner et al. 53). *Flavobacterium*,* Pseudomonas*,* Enterobacteriacae,* or *Clostridium* are known to be pathogenic bacteria commonly colonizing digestive tract of fish (Sugita et al. 91; Nayak 65). *Enterobacteriacae* recorded in one population, Le, can be a major disease agent in fish and is probably of anthropogenic origin (Newton et al. 67) and Le is a human managed freshwater location. ADONIS and CCA presented contrasted results suggesting that pathogen richness and distribution differ but not their composition. Also, the environmental factors identified by the CCA to have potential influence on pathogen distribution were also found to play a role in morphological adaptation to various habitats in the European minnow (Collin and Fumagalli 16). These concordant lines of evidence suggest that European minnows are locally adapted to both abiotic and biotic factors in their respective environment.

### Pathogen-mediated selection on MHCIIB loci

In the present study, we found that DAB1 amino acid polymorphism at PBR sites was not associated to pathogen richness, but patterns of differentiation at neutral loci and DAB1 were significantly correlated. These results could suggest an effect of migration and drift on DAB1 diversity. However, contrary to expectations where neutral processes alone were explaining genetic structure at DAB1, the pattern of isolation by distance at DAB1 remained significant even when controlling for neutral genetic structure. These results suggest (1) a strong spatial structure between populations and (2) a role for diversifying selection, with selection on DAB1 favoring different, locally adapted alleles in every population. DAB1 genetic diversity seems thus to be the result of diversifying selection promoting different locally adapted alleles frequencies in different populations. Similar results were observed on MHCIIB in recent studies on great snipes (*Gallinago media*; Ekblom et al. 27) and MHC class I in house sparrow (*Passer domesticus*; Loiseau et al. 57).

Contrarily to DAB1*,* a significant positive relationship between diversity of potentially pathogenic bacteria and DAB3 amino acid polymorphism was found. Also, patterns of genetic differentiation at DAB3 and neutral markers were not correlated, implying that selection shaped DAB3 diversity. The latter results suggest a role for contemporary pathogen-mediated selection in promoting local adaptation in terms of genetic diversity of *P. phoxinus* at DAB3. Pathogen-mediated selection linked to habitat heterogeneity has been proposed as a major mechanism shaping MHC variation (Blais et al. 10; Ekblom et al. 27; Alcaide et al. 2). In fish, there are numerous empirical examples suggesting that pathogen-mediated selection is acting to promote MHC diversity through three main mechanisms (reviewed in Wegner 101 and Eizaguirre and Lenz 23): (1) heterozygote advantage (Rakus et al. 75; Evans and Neff 29; Kekäläinen et al. 44), (2) frequency-dependent selection (Langefors et al. 52; Croisetière et al. 18; Dionne et al. 20; Eizaguirre et al. 24) and/or (3) habitat heterogeneity (Dionne et al. 19, 20; Eizaguirre et al. 25, 26). MHC differentiation observed at DAB3 is likely to be a result of habitat heterogeneity and local adaption to the local pathogen diversities. Several lines of evidence suggest that pathogen-mediated selection promotes and maintains genetic diversity at DAB3. First, IBD became only apparent for DAB3 when excluding three outlier pairs of populations and was absent otherwise. This highlights a limited role for distance-dependent gene flow in shaping the distribution of DAB3 alleles. Significant IBD after the exclusion of the three outlier pairs of populations suggests that selection (local adaptation) at DAB3 has driven allele frequency evolution in a number of populations, as for DAB1. As the outliers show low genetic differentiation at large distances, selection appears to have favored similar alleles in isolated populations. We found a significant relationship between pathogen richness and DAB3 amino acid polymorphism at PBR sites. Overall, these results suggest that the genetic diversity observed at DAB3 may be adaptive at a local scale, and that bacterial pathogen diversity represents a main selective agent. Though, we cannot exclude that only balancing selection may act on the three outlier pairs of populations at DAB3 loci. Indeed, as mentioned earlier, balancing selection usually produces pattern low of genetic differentiation at MHC loci compared to neutral markers (Hedrick et al. 38; Bernatchez and Landry 9; Seddon and Ellegren 79; Van Oosterhout 97; Evans et al. 30; Miller et al. 61). For example, Van Oosterhout et al. (98) found lower levels of genetic differentiation at MHC loci than at neutral markers in the *Poecilid* fish *Poecilia reticulata*. They explain such a pattern by MHC loci being under balancing selection, increasing the effective migration rate for these genes compared to what is expected at neutral markers. Finally, the pattern of genetic differentiation observed between populations of European minnows and the presence of outlier pairs of populations could suggest an effect of fish stocking. We can rule out this hypothesis with high confidence, as the levels of genetic differentiation for outlier pairs of population are higher at AFLP loci and DAB1 than they are at DAB3 (gene flow from stocking would lead to genome-wide low differentiation).

The slight contrasting pattern of differentiation between DAB1 and DAB3 suggests that the evolution of these two duplicates is driven by different selective agents (Landry and Bernatchez 51) and/or different pathogen-mediated selection intensity (Edwards and Hedrick 22; Martinsohn et al. 58; Penn and Potts 71; Van Oosterhout 97). Contrasting patterns of evolution of the MHCIIB duplicates similar to the ones identified here in *P. phoxinus* have already been demonstrated in other cyprinid species (Seifertova and Simkova 80). For example, chubs (*Squalius cephalius*) display a similar MHCIIB architecture with each two of the duplicates present in European minnows highlighted in the present study. In *S. cephalus* DAB3 also presents a greater diversity than DAB1. Although these data support the results found in the present work, several studies on other cyprinid fish reported the inverse pattern (Ottova et al. 69, 70).

### Combining molecular evolution and environmental genomics

Our study used a combination of molecular evolution and environmental genomics to understand how pathogenic bacteria communities and their environmental characteristics influence MHCIIB genetic diversity in nine populations of European minnow. We also used genome-wide neutral AFLP markers to compare levels of genetic differentiation at both neutral and selected markers. This study attempted to (1) characterize MHCIIB in *P. phoxinus* and (2) understand how pathogen-mediated selection shapes diversity at two MHCIIB copies. Despite providing important insights into these questions, our study also shows the technical limitations of this approach.

First, the bacteria diversity survey was based on water samples collected from a single water sampling event. Even though this may represent some limitation, this approach gave some important information about pathogen distribution and abundance that can be a starting point for further studies on European minnows or other species. Indeed, most studies aiming at detecting pathogen-mediated selection, often do not report diversity, spatial distribution, or abundance of pathogens (Wegner 101). Even though in the present study potentially pathogenic bacteria were recovered from water samples and not from fish, numerous studies on bacteria associated with fish demonstrated that gut and gill microflora (including potential pathogens) is colonized by free living aquatic bacteria and/or ones associated to food (Nayak 65; Mouchet et al. 62). Further work on the topic should also consider temporal distribution of pathogens, as time scale can be of particular importance in shaping MHC diversity.

We demonstrated that both copies presented historical evidence for positive selection, but only one copy (DAB3) presented evidence for contemporary pathogen-mediated selection. DAB1 may also be subjected to this type of selection, but as fish and water samples have been sampled during the summer, we can hypothesize that this copy may be involved in the recognition of pathogens that were not sampled at that time of the year, or other pathogen taxa.

Our next-generation sequencing of MHCIIB copies presents several limits as compared to more classical approaches. The major limitation of our approach was the population-level tagging rather than individual-based sequencing. Even though it provided a rapid and cheap way to sequence a large number of individuals, the frequency of the identified variants could not be reliably inferred. Several hypotheses related to pathogen-mediated selection could not be tested such as a potential heterozygote advantage or frequency dependent selection. Nevertheless, several recent publications successfully used a population-based 454 sequencing approach similar to the one presented in our study to assess population-level MHC variation (Spurgin et al. 89; Radwan et al. 74; Sepil et al. 82). In addition, we think that the technological progress in tagging possibilities and the longer read length now available with next-generation sequencing will soon unravel the limitations mentioned above.

## Conclusion

Duplication events and the fate of duplicates in the face of both neutral processes and selection are still sparsely understood. In this context, the present study demonstrates how host–pathogen interactions can be the driver of genetic diversity in immune-related genes, and how alternative selective regimes may influence the processes driving the evolution of gene duplicates within the same species. Further studies with higher technical resolution examining the duplication history of MHC genes in cyprinid fish in Europe and the origin of duplicates' diversity should provide new insights into the relative role of historical and contemporary selection, and neutral processes on host–pathogen related local adaptation.

## Appendix 1

Description of the 454 sequencing run details, before read sorting and classification of the two MHC classIIB exon 2 and 16S rDNA sequences

**Table:**

Amplicon	Number of reads	Mean read length	Minimum read length	Maximum read length	Mean quality score	Minimum quality score	Maximum quality score
16S rDNA	548	245	27	655	27	16	40
DAB1	7591	275	15	685	28	11	40
DAB3	7877	274	18	768	28	11	40

## Supporting Information

**Figure S1.** Schematic illustration of the *Phoxinus phoxinus* MHCIIB duplicates DAB 1 (A) and DAB 3 (B). Shaded boxes represent exons. The positions of the primers used in this study are indicated by arrows.

**Figure S2.** Details on sequence reads and quality scores from the 454 run. (A) Histogram showing sequence read quality score frequencies for all reads, bacteria 16S rDNA reads, DAB1 and DAB3 reads. Red line indicates the average quality score per class of reads (B) Histogram showing sequence read length frequencies for all reads, bacteria 16S rDNA reads, DAB1 and DAB3 reads. Red line indicates the average quality score per class of reads (C) Correlation between reads qualities and reads length for each class of reads. Red lines represent the least square regression between read quality and length. R statistics from correlation Pearson tests and their significance (*** significant at 99% level, ns nonsignificant at the 99% level) are indicated.

**Table S1.** List of primers used to (A) characterize MHC Class II B in *Phoxinus phoxinus*; (B) amplify bacterial 16S rDNA and genotype MHC Class II B in P. phoxinus.
