# Supplementary figures and images for: Combining molecular evolution and environmental genomics to unravel adaptive processes of MHC class IIB diversity in European minnows (Phoxinus phoxinus)
Source: Ecol Evol. 2013 Jun 28;3(8):2568–85. doi: 10.1002/ece3.650 (PMC3930049; doi:10.1002/ece3.650)

A

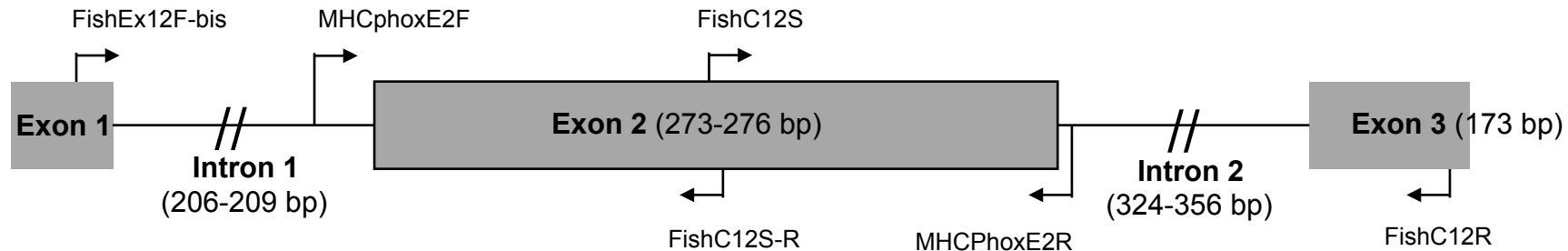

B

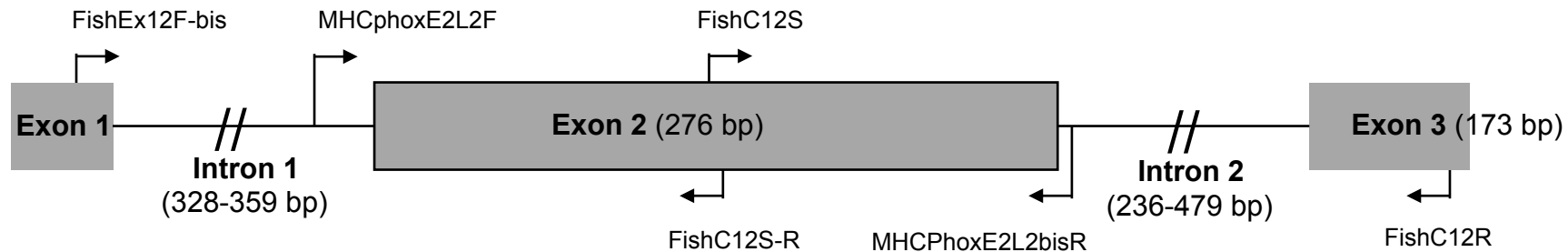

Supplement: Supplementary file 1 — Figure S1. Schematic illustration of the Phoxinus phoxinus MHCIIB duplicates DAB 1 (A) and DAB 3 (B). Shaded boxes represent exons. The positions of the primers used in this study are indicated by arrows. [file ece30003-2568-SD1.pdf]

A

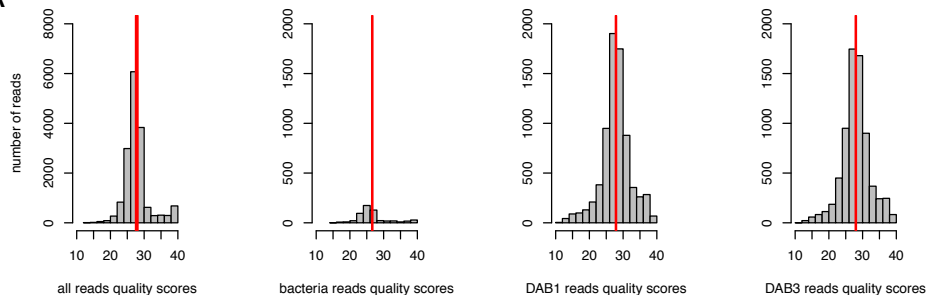

B

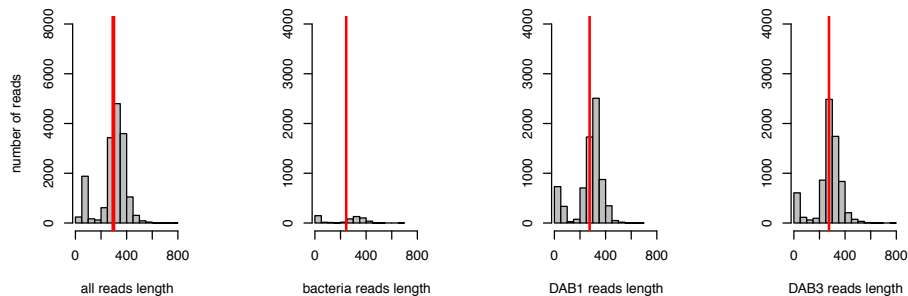

C

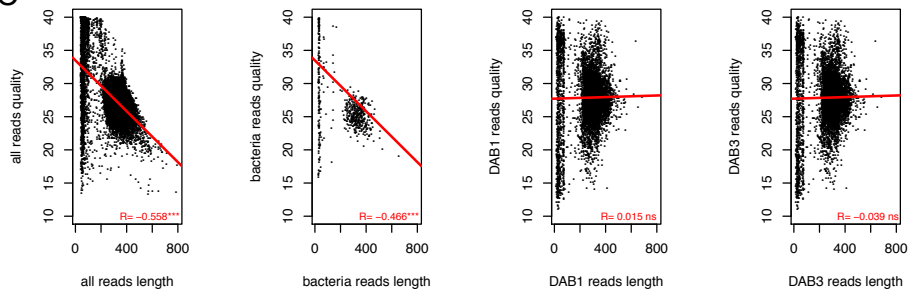

Supplement: Supplementary file 2 — Figure S2. Details on sequence reads and quality scores from the 454 run. (A) Histogram showing sequence read quality score frequencies for all reads, bacteria 16S rDNA reads, DAB1 and DAB3 reads. Red line indicates the average quality score per class of reads (B) Histogram showing sequence read length frequencies for all reads, bacteria 16S rDNA reads, DAB1 and DAB3 reads. Red line indicates the average quality score per class of reads (C) Correlation between reads qualities and reads length for each class of reads. Red lines represent the least square regression between read quality and length. R statistics from correlation Pearson tests and their significance (*** significant at 99% level, ns nonsignificant at the 99% level) are indicated. [file ece30003-2568-SD2.pdf]
